# Pylephlebitis Secondary to Diverticulitis Diagnosed by Abdominal Ultrasound and Computed Tomography

**DOI:** 10.7759/cureus.73358

**Published:** 2024-11-09

**Authors:** Takuya Maejima, Etaro Hashimoto, Kazuhito Hirose, Kenji Miyazaki, Masatsune Suzuki, Tetsuhiro Maeno

**Affiliations:** 1 Department of General Medicine, Kasama City Hospital, Kasama, JPN; 2 Department of Primary Care and Medical Education, Faculty of Medicine, University of Tsukuba / University of Tsukuba Hospital, Tsukuba, JPN; 3 Department of General Medicine, Tsukuba Medical Center Hospital, Tsukuba, JPN

**Keywords:** computed tomography (ct), portal vein pylephlebitis, septic shock (ss), small bowel diverticulitis, trans-abdominal ultrasound

## Abstract

Pylephlebitis is a rare infection, characterized by non-specific symptoms such as abdominal pain, that often leads to delayed diagnosis, yet it is a severe infection with a high mortality rate. Imaging studies are essential for diagnosis, and contrast-enhanced abdominal CT and abdominal ultrasound are commonly performed.

A 51-year-old male was admitted to the hospital with fever and shock. Blood tests revealed liver and kidney dysfunction. Contrast-enhanced computed tomography (CT) is the best modality to demonstrate the portal vein abnormalities and diverticulitis. But plain abdominal CT was performed due to renal impairment, revealing findings suggestive of small bowel diverticulitis and paralytic ileus. Septic shock, presumably caused by bacterial translocation secondary to paralytic ileus, was diagnosed, and meropenem was initiated. Subsequent abdominal ultrasound revealed thrombosis in the portal vein and an abscess in the region suspected of being affected by small bowel diverticulitis. Based on these findings, it was concluded that the patient developed pylephlebitis, septic shock, and paralytic ileus as a result of small bowel diverticulitis and the associated abscess formation. Oral amoxicillin/clavulanate was continued until the abscess resolved.

Pylephlebitis often progresses to septic shock, as seen in the present case. In severe cases of intra-abdominal infections, such as diverticulitis, it is necessary to consider the possibility of pylephlebitis and actively perform imaging studies to confirm the diagnosis. Additionally, in cases where contrast-enhanced CT cannot be performed, abdominal ultrasound is useful for diagnosis.

## Introduction

Pylephlebitis often presents with non-specific symptoms, leading to frequent delays in diagnosis [1]. On the other hand, it often progresses to septic shock and remains a serious infection with a mortality rate of 8.7-32% [2-5]. The incidence of pylephlebitis is rare, ranging from 0.37 to 2.7 cases per 100,000 person-years [2]. Intra-abdominal infections, such as diverticulitis and appendicitis, are considered to be the most common causes of pylephlebitis [2]. The portal vein drains blood from nearly all segments of the intestines, with the exception of the lower rectum. Pylephlebitis begins with thrombophlebitis of small veins originating from an infected area of the intestines and thrombophlebitis extends into the portal vein [2]. This explains why diverticulitis and appendicitis account for a large proportion of pylephlebitis cases. Imaging studies are essential for the diagnosis of pylephlebitis. Contrast-enhanced abdominal computed tomography (CT) is able to reveal findings suggestive of pylephlebitis [6,7] and provides comprehensive evaluation of the entire abdominal cavity. Abdominal ultrasound may also be performed as an alternative modality [8]. A treatment duration of approximately 4 to 6 weeks of antibiotic administration is required [2]. Since pylephlebitis often requires a longer duration of antibiotic therapy compared to uncomplicated intra-abdominal infections such as diverticulitis, it is important to evaluate the presence of this complication.

In this case report, we present a patient with small bowel diverticulitis complicated by septic shock. Although contrast-enhanced abdominal CT could not be performed due to acute kidney injury, the diagnosis of pylephlebitis and a peridiverticular abscess was made using abdominal ultrasound, and appropriate treatment was administered. Small bowel diverticulitis is a rare infection, and its treatment is considered similar to that of colonic diverticulitis [9]. There are no reported cases of small bowel diverticulitis complicated by pylephlebitis. This report highlights the importance of searching for severe complications that may alter treatment management, even in conditions with a generally favorable prognosis, such as diverticulitis. It also emphasizes the importance of proactive diagnosis using abdominal ultrasound in situations where contrast-enhanced abdominal CT cannot be performed for the detection of pylephlebitis.

## Case presentation

A 51-year-old male was admitted to the hospital with fever and shock. The patient developed a fever the day before admission and visited a previous physician on the day of admission, where he was found to be in a state of shock and was transferred to our hospital. He had a medical history of dilated cardiomyopathy and chronic kidney disease. He had no history of small bowel diverticula. He had no allergies and did not consume alcohol or smoke.

Upon arrival, his consciousness level was a Glasgow Coma Scale (GCS) score of E4V5M6. His vital signs were as follows: body temperature 38.8°C, blood pressure 53/34 mm Hg, heart rate 106 bpm, respiratory rate 32 bpm, and SpO2 98% on 10 L of oxygen administered via a reservoir mask. Large-volume intravenous fluid resuscitation was initiated immediately. However, blood pressure could not be maintained. Subsequently, norepinephrine and vasopressin were administered, leading to the stabilization of blood pressure.

**Figure 1:**
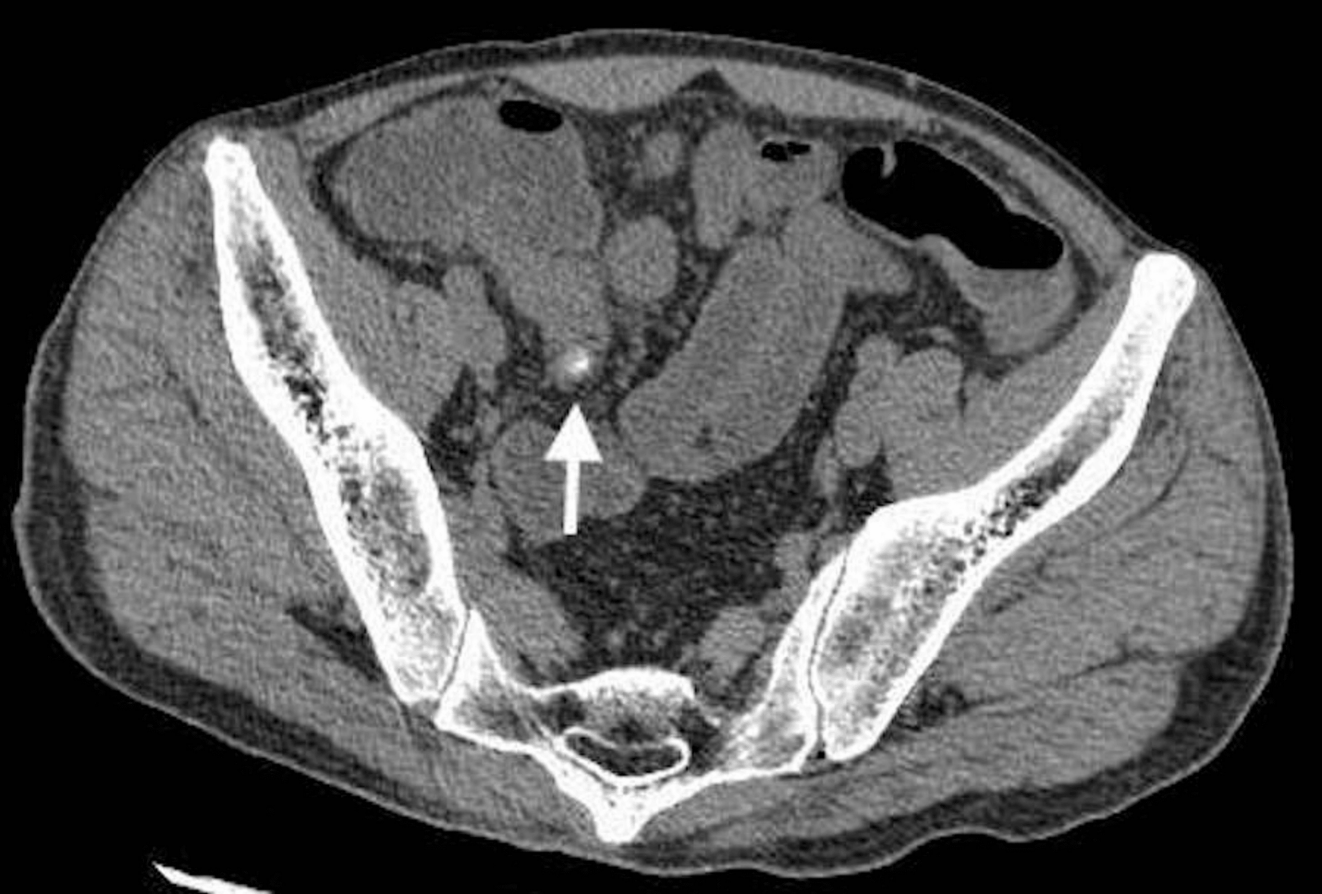
Calcified diverticula in the ileum (arrow)

**Figure 2:**
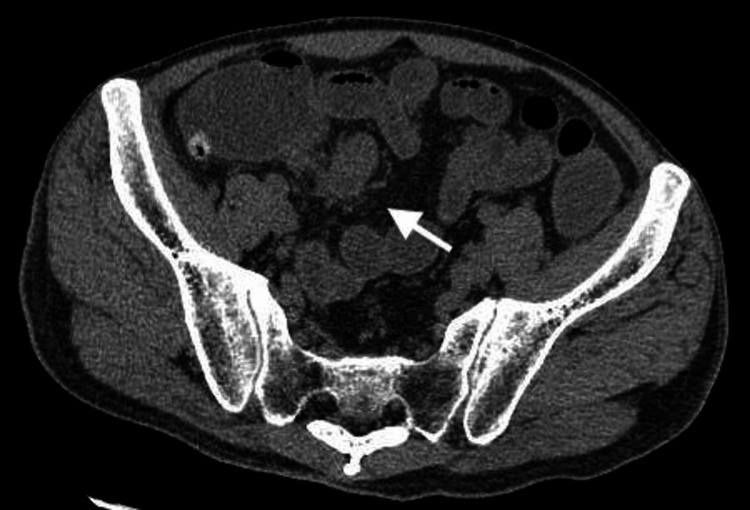
Subtle increase of fat tissue density in the surrounding area of diverticula (arrow)

**Figure 3:**
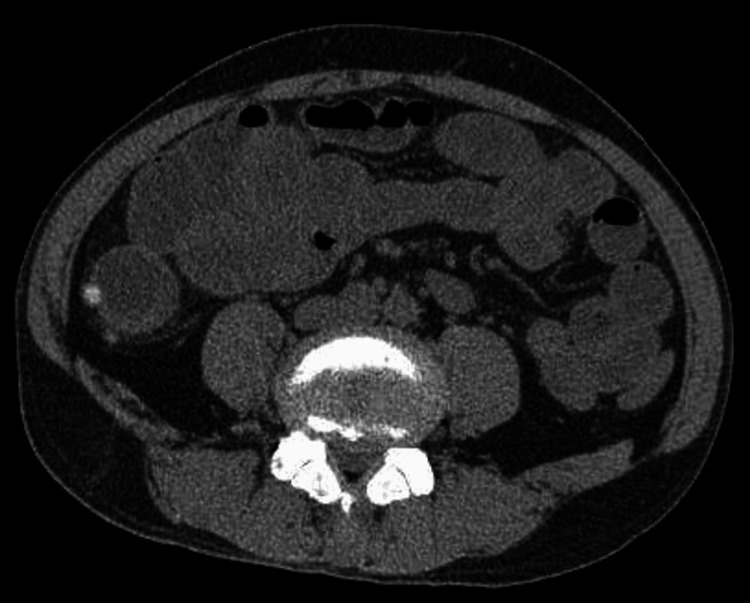
Generalized bowel dilatation

Blood tests revealed elevated inflammatory markers, elevated liver enzymes, and renal impairment. Blood gas analysis revealed a lactate level of 7.87 mmol/L (Table 1). Urinalysis did not show any pyuria (Table 2). Due to the unknown baseline renal function and the presence of severe renal impairment at the time of admission, the use of contrast agents was avoided. An abdominal plain CT scan was performed, which revealed diverticula in the ileum, with surrounding fat stranding noted, raising suspicion of diverticulitis (Figures 1, 2). Additionally, there was dilatation of the bowel proximal to the same site, while intestinal fluid was observed in the distal segment, suggesting a state of paralytic ileus (Figure 3). Based on the above findings, it was concluded that the patient had progressed to septic shock due to bacterial translocation associated with paralytic ileus, and treatment with meropenem was initiated. Although the cause of the ileus was unclear, a nasogastric tube was placed to attempt decompression of the bowel. Comprehensive management was initiated in the intensive care unit.

**Table 1:** Blood test

Laboratory tests	Results	Normal range
White blood cell counts (/μL)	13900	3300-8600
Hemoglobin (g/dL)	12.1	13.7-16.8
Platelet count (x10^3^/μL)	143	158-348
Aspartate aminotransferase (U/L)	269	13-30
Alanine aminotransferase (U/L)	302	10-42
Total bilirubin (mg/dL)	5.9	0.4-1.5
Alkaline phosphatase (U/L)	206	38-113
γ-glutamyl transpeptidase (U/L)	204	13-64
Lactate dehydrogenase (U/L)	261	124-222
Creatinine (mg/dL)	3.04	0.65-1.07
Blood urea nitrogen (mg/dL)	40.5	8.0-20.0
C-reactive protein (mg/dL)	12.87	<0.14
Activated partial thromboplastin time (sec)	36.9	24-39
Prothrombin time (% in the normal range)	38	80-100
Potentiel hydrogen (pH)	7.354	7.35-7.45
Lactate (mmol/L)	7.87	0.50-2.00
Bicarbonate (mmol/L)	23.0	20.0-26.0

**Table 2:** Urine dipstick analysis

Laboratory tests	Results	Normal range
Leukocyte esterase	-	-
Occult hematuria	2+	-
Nitrite	-	-
Protein	2+	-
Glucose	-	-

On the 2nd day of hospitalization, intestinal bacteria were isolated from two sets of blood cultures obtained on admission, which was considered consistent with bacterial translocation. On the 3rd day of hospitalization, the blood culture results revealed that the isolate was E. coli, which exhibited good sensitivity to antibiotics. Consequently, the treatment was changed to ceftriaxone. The treatment course was favorable, and on the 4th day of hospitalization, the vasopressor was discontinued.

**Figure 4:**
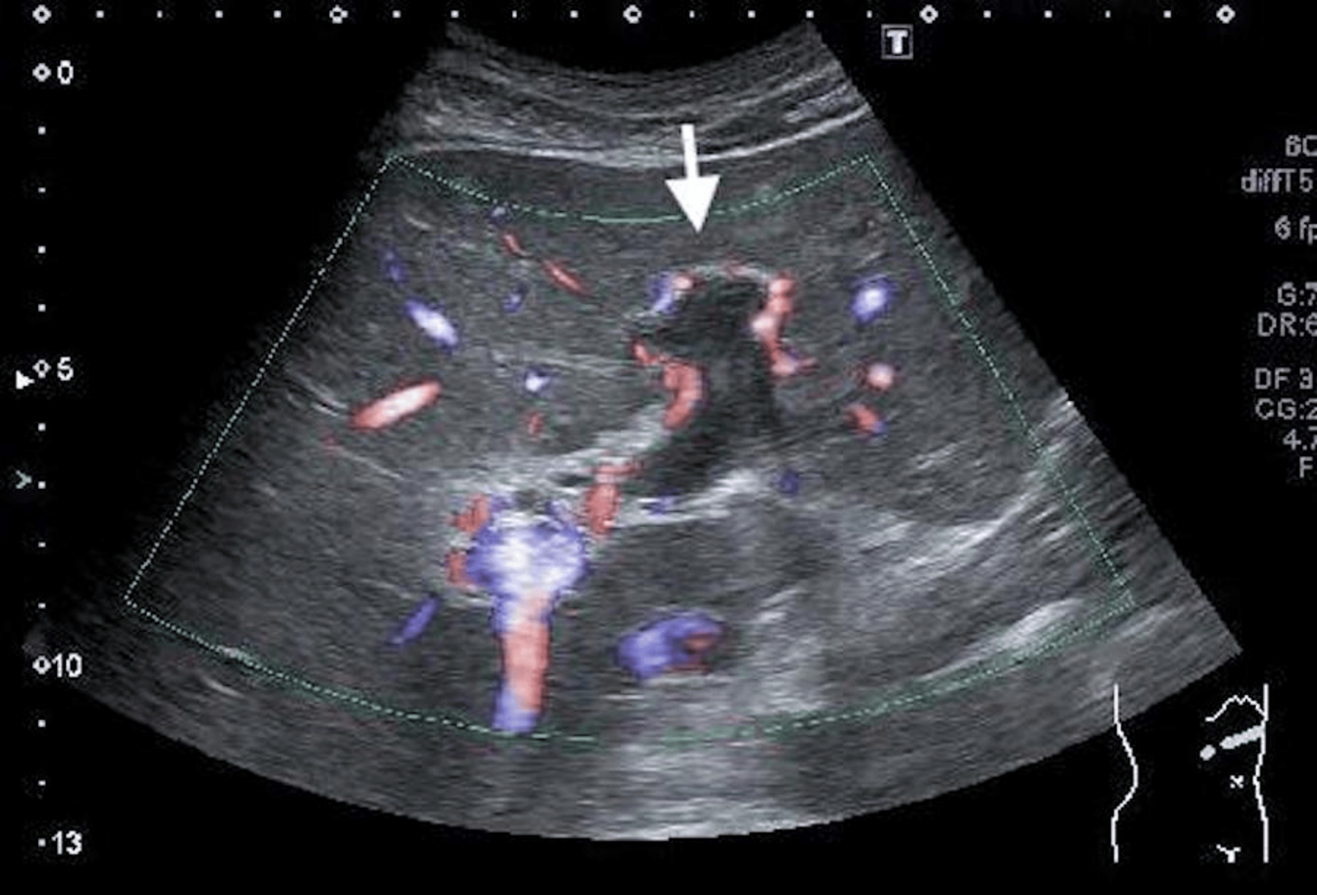
A thrombus extending from the left branch of the portal vein to the branches of the left lobe of the liver (arrow)

**Figure 5:**
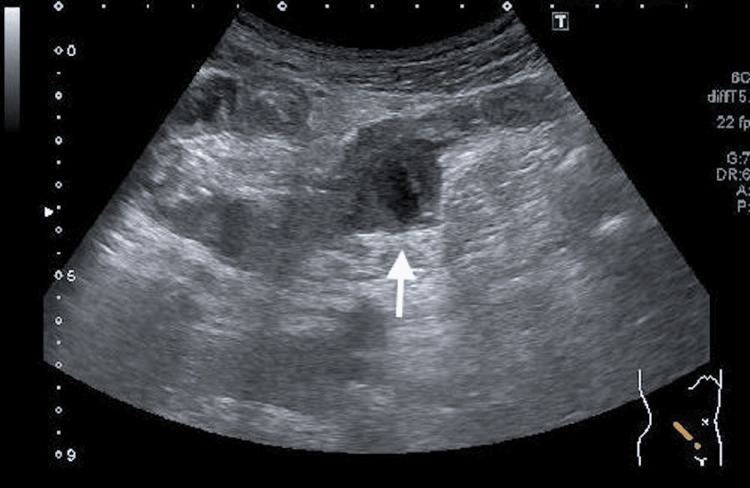
Abscess formation at the site of diverticulitis observed on the plane CT (arrow)

**Figure 6:**
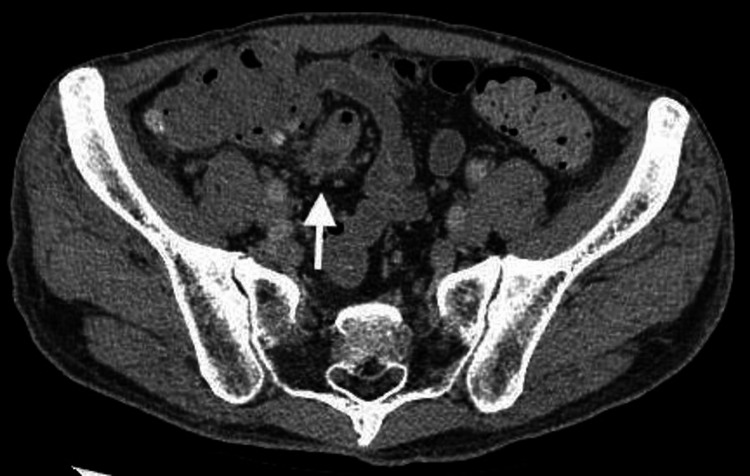
Approximately 2 cm abscess (arrow) This corresponds to the location indicated by the arrow in Figure 2, which could not be identified as an abscess on the plain CT.

**Figure 7:**
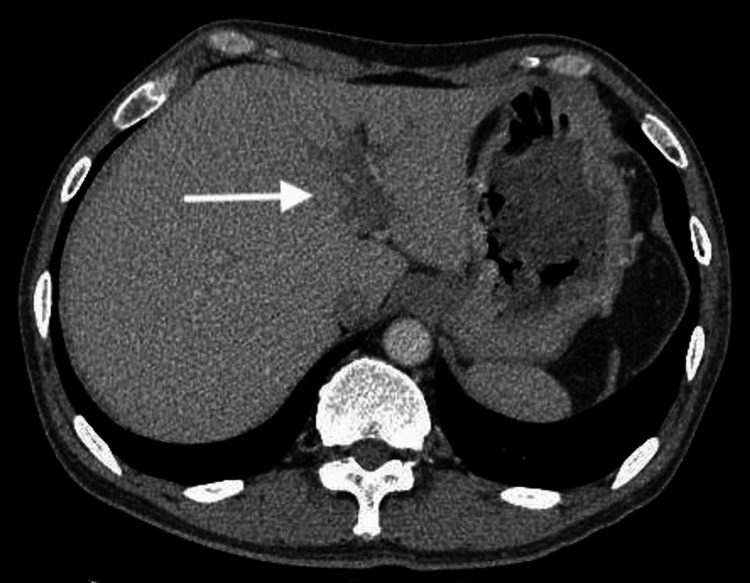
Thrombus of the left branch of the portal vein (arrow)

**Figure 8:**
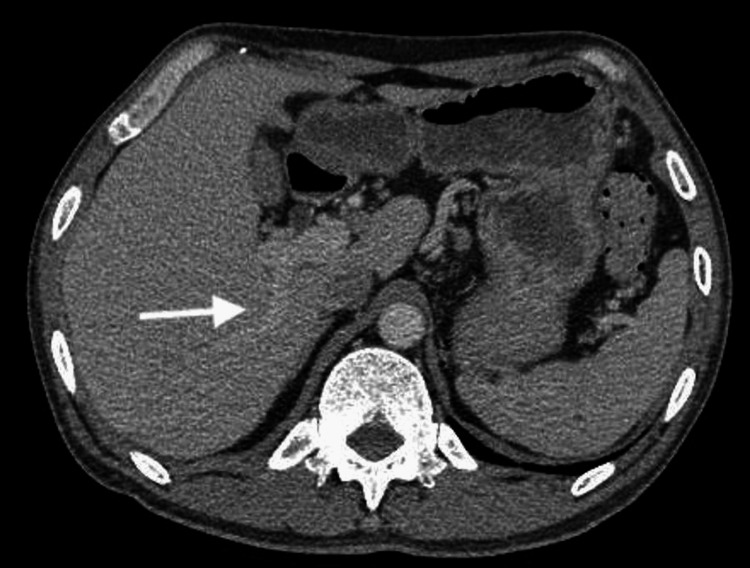
Right branch of the portal vein (arrow) For reference, the contrast-enhanced right branch of the portal vein is also presented.

To further investigate the causes of septic shock and ileus, a contrast-enhanced CT scan was considered. However, due to insufficient improvement in renal function, an abdominal ultrasound was requested from the radiologist on the 10th day of hospitalization. Thrombosis was observed from the left branch of the portal vein to the branches of the left lobe of the liver (Figure 4), and there were findings suggesting abscess formation at the site of diverticulitis identified on the plain CT scan (Figure 5). Thus, it was considered that small bowel diverticulitis, along with the formation of a surrounding abscess, led to the development of pylephlebitis, septic shock, and paralytic ileus. Therefore, the decision was made to continue antibiotic therapy for a longer duration, and in this case, given the presence of abscess formation, treatment would be continued until the abscess had disappeared. After waiting for improvement in renal function, a contrast-enhanced CT scan was performed on the 21st day of hospitalization, revealing an approximately 2 cm abscess adherent to the bowel at the site of diverticulitis (Figure 6). Although the portal vein thrombosis was still present (Figures 7, 8), there were no findings indicating exacerbation compared to the abdominal ultrasound results. Additionally, no findings suggestive of gastrointestinal infections in areas other than the diverticulitis site, nor any indications of intestinal ischemia, were observed. On the same day, the blood test showed a decrease in the white blood cell count to 4,700 /μL, and the C-reactive protein (CRP) level had also fallen to 0.35 mg/dL. On the 24th day of hospitalization, the patient was discharged, and the antibiotic regimen was switched to oral amoxicillin/clavulanate.

The patient was followed up in the outpatient clinic, and blood tests conducted 12 days after discharge showed a white blood cell count of 6,100 /μL and a CRP level of 0.04 mg/dL. On the abdominal contrast-enhanced CT conducted 28 days after discharge, an abscess was still present. Additionally, while the portal vein thrombosis was still present, it showed a tendency to decrease in size when compared to the CT scan conducted on the 21st day of hospitalization. On the abdominal contrast-enhanced CT conducted 97 days after discharge, the abscess had scarred. Therefore, the antibiotic treatment was discontinued at that time. The portal vein had atrophied, and the thrombus had also become organized.

## Discussion

This case illustrates two important learning points. First, in patients with diverticulitis, especially those with severe complications such as septic shock, it is necessary to suspect pylephlebitis. Second, abdominal ultrasound is also effective for the diagnosis of pylephlebitis, and diagnosis can be made even in situations where a contrast-enhanced abdominal CT scan cannot be performed.

While diverticulitis itself is a very common infection, pylephlebitis is considered a rare infection. The reported incidence of pylephlebitis complicating colonic diverticulitis is 3% [10]. Due to the rarity of small bowel diverticulitis, the incidence of complications such as pylephlebitis is not well established. In cases of pylephlebitis caused by Fusobacterium necrophorum, there is a report indicating that all patients experienced right upper quadrant pain and fever [11]. However, pylephlebitis, including cases caused by E. coli, typically presents with symptoms such as fever (75.5%) and abdominal pain (66.4%), as well as other symptoms such as nausea and chills [2]. These symptoms often overlap with those of underlying conditions causing pylephlebitis, such as diverticulitis or appendicitis. Moreover, the symptoms of these underlying conditions are sometimes subtle, which frequently leads to delayed diagnosis [1]. Blood tests show an increase in white blood cell count (89.7%), elevated CRP levels (90.9%), elevated bilirubin levels (74.6%), and elevated aspartate transaminase/alanine transaminase (AST/ALT) levels (71.6%) [3]. In this case, elevations in bilirubin and transaminases were also observed, and these findings may serve as supportive evidence for the diagnosis of pylephlebitis. For diagnosis, abdominal contrast-enhanced CT or abdominal ultrasound is performed. Abdominal contrast-enhanced CT reveals a filling defect in the portal vein [2]. Abdominal ultrasound shows the presence of echogenic material within the portal vein, accompanied by the absence of color Doppler signals in that area [2]. There have been no reports stating that diagnosis was made using plain abdominal CT. In this patient, the absence of abdominal pain despite noted liver dysfunction, along with the inability to perform abdominal contrast-enhanced CT due to renal impairment, may have contributed to the delay in diagnosis. Fortunately, in this case, the antibiotic treatment was effective, leading to a relatively prompt improvement from the state of septic shock. However, considering the high mortality rate and the possibility of prolonged antibiotic treatment, it is crucial to include pylephlebitis in the differential diagnosis for patients with diverticulitis complicated by septic shock, despite its rarity.

In situations where performing contrast-enhanced CT is difficult, as in this case, it is important to perform abdominal ultrasound for the diagnosis of pylephlebitis. According to the previous report [3], abdominal CT was performed in 89.3% of patients with pylephlebitis, while abdominal ultrasound was performed in 38.8% of patients. There are no studies that have evaluated the sensitivity and specificity of each modality for pylephlebitis, making it difficult to determine which examination is superior for diagnosis. Because contrast-enhanced CT can evaluate the entire abdominal cavity, it is capable of diagnosing conditions such as diverticulitis and appendicitis, which may be the underlying causes of pylephlebitis. On the other hand, abdominal ultrasound is easier to perform, even in cases like this patient where there is impaired renal function, and can be conveniently performed at the bedside. However, it is difficult to visualize the entire abdominal cavity, and the effectiveness of the examination depends on the skill of the operator. Furthermore, there are reports that abdominal ultrasound has failed to diagnose pylephlebitis [12, 13]. Nevertheless, in some cases, as in the present case, abdominal ultrasound can be used to diagnose pylephlebitis. Considering that ultrasound can be easily performed, we believe that it should be performed actively.

## Conclusions

In cases of diverticulitis accompanied by severe complications such as septic shock, pylephlebitis should be included in the differential diagnosis, and imaging studies should be actively performed. Elevated bilirubin and transaminases serve as a rationale for a more aggressive search for pylephlebitis. If contrast-enhanced CT cannot be performed due to renal dysfunction, abdominal ultrasound is a useful diagnostic tool.
